# Rope Tension Fault Diagnosis in Hoisting Systems Based on Vibration Signals Using EEMD, Improved Permutation Entropy, and PSO-SVM

**DOI:** 10.3390/e22020209

**Published:** 2020-02-12

**Authors:** Shaohua Xue, Jianping Tan, Lixiang Shi, Jiwei Deng

**Affiliations:** 1 School of Mechanical and Electrical Engineering, Central South University, Changsha 410010, China; xueshaohua@csu.edu.cn (S.X.); 173701022@csu.edu.cn (L.S.); dengjw@csu.edu.cn (J.D.); 2 State Key Laboratory of High Performance and Complex Manufacturing, Changsha 410010, China

**Keywords:** rope tension, fault diagnosis, permutation entropy, EEMD, PSO-SVM

## Abstract

Fault diagnosis of rope tension is significantly important for hoisting safety, especially in mine hoists. Conventional diagnosis methods based on force sensors face some challenges regarding sensor installation, data transmission, safety, and reliability in harsh mine environments. In this paper, a novel fault diagnosis method for rope tension based on the vibration signals of head sheaves is proposed. First, the vibration signal is decomposed into some intrinsic mode functions (IMFs) by the ensemble empirical mode decomposition (EEMD) method. Second, a sensitivity index is proposed to extract the main IMFs, then the de-noised signal is obtained by the sum of the main IMFs. Third, the energy and the proposed improved permutation entropy (IPE) values of the main IMFs and the de-noised signal are calculated to create the feature vectors. The IPE is proposed to improve the PE by adding the amplitude information, and it proved to be more sensitive in simulations of impulse detecting and signal segmentation. Fourth, vibration samples in different tension states are used to train a particle swarm optimization–support vector machine (PSO-SVM) model. Lastly, the trained model is implemented to detect tension faults in practice. Two experimental results validated the effectiveness of the proposed method to detect tension faults, such as overload, underload, and imbalance, in both single-rope and multi-rope hoists. This study provides a new perspective for detecting tension faults in hoisting systems.

## 1. Introduction

Hoisting systems are widely applied in various industries, especially the mining industry. A mine hoist is a piece of key equipment for transporting ore, materials, and workers between the surface and underground. Hoisting containers are connected to hoist drums by wire ropes that are thousands of meters long, which means that rope tension plays a significant role in hoisting safety. Tension faults such as overload, underload, and imbalance are serious threats to hoisting safety and production. Therefore, it is of considerable significance to diagnose tension faults in hoisting systems [1].

**Figure 1:**
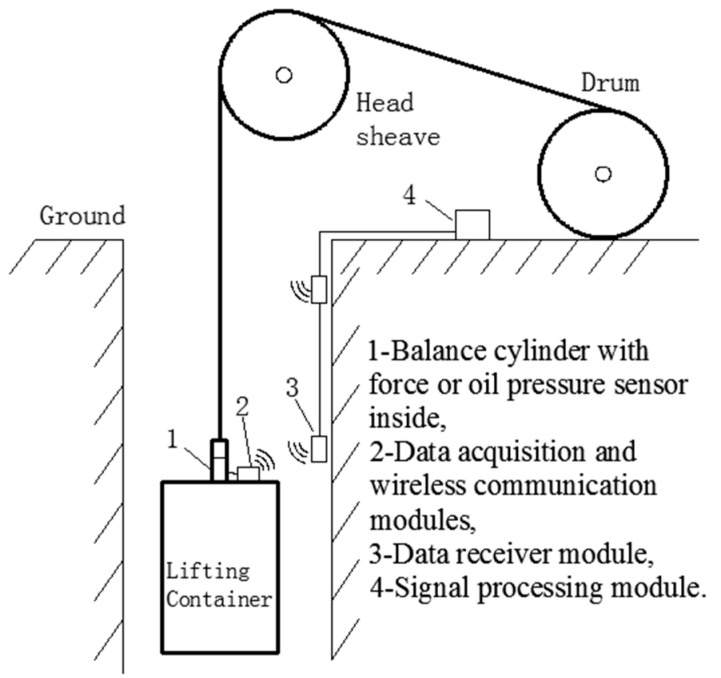
Schematic diagram of conventional tension fault detection in a hoisting system.

Conventional tension condition monitoring is based on force sensors or oil pressure sensors, which are installed at the connection between wire ropes and lifting containers [2]. The data are transmitted through a long mine shaft to the ground by wireless communication, as shown in Figure 1. However, this method faces several challenges. First, underground environments are very harsh because of spray water, dust, and corrosive gas, resulting in low reliability of the measuring devices. Second, power supply and data transmission in a moving cage are inconvenient. Moreover, the installation of force sensors may negatively impact the original structure, which leads to hidden danger and safety regulation violations [3].

Mechanical vibration signals often contain sufficient information on the running state of mechanical equipment, so they are widely applied in fault diagnosis of gears, motors, and bearings [4,5,6,7]. Compared with the force-sensor based method, the vibration-based method has obvious advantages. First, vibration sensors are easy to install and do not damage the safety of the original structure. Second, vibration signals react immediately to changes. Third, processing techniques for vibration signals are abundant [7]. Therefore, the vibration-based method provides a potential approach to diagnose rope tension faults in hoisting systems [8]. 

Vibration signal processing usually consists of three steps, namely signal decomposition, feature extraction, and pattern recognition. Common signal decomposition methods include Fourier transform (FT)-based methods, wavelet transform (WT)-based methods, and empirical mode decomposition (EMD)-based methods [7,9]. The FT-based method is a traditional method to convert the time domain to the frequency domain. However, it is not suitable for non-stationary signals, which are quite common in the mining industry [10]. Although short-time Fourier transform (STFT) improves the FT method, it also has obvious limitations because the shape and size of the window are fixed for all frequencies [11]. The WT-based method can decompose a non-stationary signal into a set of basis functions consisting of contractions, expansions, and translations of a mother function. However, the selection of the key wavelet base and decomposition level significantly relies on the user’s experience [12]. EMD is a new decomposition method [13], which can self-adaptively decompose any signal into a set of intrinsic module functions (IMFs) that include different frequency characteristics. It is a significant advance in the analysis of non-stationary signals. However, EMD has a drawback of mode mixing. To overcome this drawback, Wu and Huang proposed the ensemble empirical mode decomposition (EEMD) method, which is a noise-assisted data analysis method by adding finite white noise to the investigated signal [14]. Because of their ease of use and excellent performance for complex signals, EMD-based methods have been widely applied in fault diagnosis [15,16,17,18,19,20,21,22]. For example, Lei et al. applied the EEMD to the rub-impact fault diagnosis of a power generator and early rub-impact fault diagnosis of a heavy oil catalytic cracking machine [15]. Jaouher et al. applied the EMD to diagnose bearing faults [16]. Bustos et al. used the EMD for estimating the condition of the high-speed train running gear system [20]. Park et al. applied EEMD to the transmission error measured by the encoders of the input and output shafts to classify the spall and crack faults of gear teeth [21]. Buzzoni et al. applied EMD-based methods for the diagnosis of localized faults in multistage gearboxes, and they pointed out that EMD-based methods were particularly suitable for industrial applications since they were completely automatic [22]. Considering the nonlinearity of the signal in real hoists and the industrial ease of use, EEMD is selected in this paper. To improve the diagnosis accuracy, the selection of the main IMFs is necessary since not all IMFs are sensitive to the faults [23]. Some selection criteria have been proposed, such as the correlation coefficient criterion [22], the kurtosis criterion [24], and the similarity between the probability density function of the raw signal and that of each IMF [23]. However, they were all designed for rigid systems, especially bearings. 

The effectiveness of fault diagnosis largely depends upon the quality of extracted features [25]. The earliest features are mainly statistical parameters such as kurtosis, root mean square, mean etc. However, they are often ineffective in a complex situation. Different running states result in different dynamic characteristics, which can be described from energy and entropy. Entropy is an effective measure to characterize the complexity of time series, and has attracted wide attention in recent years. Many entropy approaches have been proposed, such as sample entropy, approximate entropy, and permutation entropy (PE) [26]. PE is a measure of the complexity in time series data based on the comparison of successive adjacent values which are mapped to ordinal patterns [27]. Compared with others, PE has apparent advantages of simple calculation and robustness to noise, thus it has been widely applied. Berger et al. applied PE in the analysis of complex electroencephalography signals [28]. Zanin et al. reviewed applications of PE in biomedical and econophysics fields [29]. PE is also used in fault diagnosis. For example, Mitiche et al. applied PE in the classification of the electromagnetic discharge states in high-voltage power generation [30]. However, the PE lost amplitude information of the original signal [31]. In [26], logistic models combining PE and sample entropy/ approximate entropy proved to be much more effective than PE in body temperature signal classification since PE contains only subpattern ordinal differences, while the combined model adds subpattern amplitude differences.

In pattern recognition, support vector machine (SVM), artificial neural network (ANN), and deep learning (DL) are attractive [32,33]. Liu et al. reviewed the applications of ANN, SVM, and DL in the fault diagnosis of rotating machinery and compared their advantages and disadvantages [32]. ANN has a good approximation of complex nonlinear function; however, many parameters need to be adjusted, and a lot of training samples are required when using ANN. DL does not need the feature extraction; however, large numbers of training samples are required. SVM has a high classification accuracy. Further, it has the advantage of solving small-sample learning problems because it is based on structural risk minimization instead of experiential risk minimization [34,35]. The performance of ANN, SVM and k Nearest Neighbor (kNN) in misfire and valve clearance faults detection in the combustion engines was compared in [36]. It showed that SVM outperformed kNN and ANN in terms of average accuracy, sensitivity, and specificity. Similar conclusion was also drawn in detecting bearing failures in wind-turbine gearboxes in [33]. Furthermore, the training and tuning time of SVM was found smaller than that of ANN since ANN has more parameters to tune. Considering the small simple problem and time consuming requirement in real hoisting systems, SVM is selected in this work. In an SVM with a Gaussian kernel, the penalty parameter *c* and the kernel parameter *g* have a great influence on the classification accuracy. However, the parameter selection lacks guidance [37]. So, some optimization algorithms, such as the genetic algorithm (GA) [38] and the particle swarm optimization (PSO) algorithm [39] have been used to find the best parameters. GA was introduced in the mid-1970s by John Holland. It is inspired by the principles of genetics and evolution and mimics the reproduction behavior observed in biological populations. PSO was proposed by Kennedy and Eberhart in the mid-1990s. It is inspired by the ability of flocks of birds to adapt to their environment by implementing an “information sharing” approach. Venter compared the PSO and the GA and pointed out that the PSO had similar effectiveness as the GA but with significantly better computational efficiency by implementing statistical analysis and formal hypothesis testing [40]. The effectiveness and high efficiency of the PSO make it applied in fault diagnosis [41,42]. For instance, in [41], PSO was the key to finding the optimized weights of damage-sensitivitive feature vectors in fault detection of bearing systems. 

Inspired by previous studies, we propose a novel method to diagnose tension faults based on the vibration signal of the head sheave. The EEMD is used for decomposition and denoising. The improved permutation entropy (IPE) is proposed for feature extraction. The PSO-SVM is employed for classification. Experimental results demonstrated the effectiveness of the proposed method to classify tension faults in both single-rope and multi-rope hoists. The remainder of this article is organized as follows: Section 2 introduces the theoretical basis and the proposed method. Section 3 presents the experimental results and discussions, and Section 4 provides the conclusion.

## 2. Theoretical Basis

### 2.1. EEMD and the Proposed Sensitivity Index to Extract the Main IMFs

The EMD method was proposed by Huang in the 1990s in order to decompose nonlinear and non-uniform signals [13]. It can adaptively decompose a complex signal into several IMFs and a residue, which represent the simple oscillatory modes and the central tendency embedded in the signal, respectively. An IMF should have the following conditions: (1) the difference between the number of extrema and the number of zero-crossings should be zero or one in the whole data set and (2) the mean value of the upper and lower envelopes is zero. The EMD process of the signal x(t) is as follows:

1. Extract the local maxima and minima of x(t).
2. Interpolate the maxima and minima by cubic spline lines to determine the upper envelope u(t) and lower envelope v(t), respectively.
3. Calculate the mean value $m_{1}(t)=(u(t)+v(t))/2$.
4. Let $h_{1}(t)=x(t)-m_{1}(t)$, if $h_{1}(t)$ is an IMF then let $c_{1}(t)=h_{1}(t)$, else regard $h_{1}(t)$ as x(t) and go back to step 1.
5. Define the residue $r_{1}(t)=x(t)-c_{1}\left(t\right)$.
6. Regard $r_{1}(t)$ as x(t), and repeat the above steps *n* times, until the residue $r_{n}$ is a monotone function or smaller than the threshold.

At the end of the procedure, a residue $r_{n}$ and *n* IMFs $c_{k}(k=1,2,...n)$ are obtained. The original signal can be described as in Equation (1):

(1) $$x(t)=\sum_{k=1}^{n}c_{k}(t)+r_{n}(t)$$

However, the EMD method has the disadvantage of mode mixing, which means that a single IMF includes oscillations of dramatically disparate scales or a similar scale component resides in different IMFs. The EEMD method is proposed to overcome this problem [14]. White noise is added to the entire time-frequency space uniformly to project the different components onto the project scales automatically. The EEMD method is executed as follows:

1. Add white noise to the original signal.
2. Decompose the noise-added signal IMFs by the EMD method.
3. Repeat steps 1 and 2 with different white noises each time.
4. Calculate the ensemble mean value of each IMF as the final IMF.

The amplitude of the added noise can be set as 0.2 times the standard deviation of the signal and the number of the ensembles can be set as 50–100, as suggested in [14].

The selection of main IMFs is necessary to improve the diagnosis accuracy because not all the composed IMFs contain useful information. Some selection criteria have been proposed, such as the correlation coefficient criterion, the kurtosis criterion; however, the valuable IMFs may be lost only based on a single dimension. Further, they are designed for the fault diagnosis of rigid bearings instead of flexible rope systems. For example, the kurtosis is sensitive to the impulses produced by rigid body collisions, which happen easily in bearings but not in flexible systems. In this paper, a sensitivity index to select the main IMFs, which considers the energy and similarity to the original signal, is proposed as

(2) $$s_{k}=a\frac{|C_{k}|}{\sum_{n}|C_{k}|}+(1-a)\frac{E_{k}}{\sum_{n}E_{k}}$$

where *n* is the decomposed number of IMFs; $E_{k}$ is the energy of the *k*-th IMF, $C_{k}$ is the Pearson correlation coefficient between the *k*-th IMF and the original signal, and a is an adjusting coefficient to make $s_{k}$ more flexible, which is in the range of [0, 1]. The Pearson correlation coefficient *C* between M-point sequences X and Y is defined in Equation (3):

(3) $$C=\frac{\sum XY-\frac{\sum X\sum Y}{M}}{\sqrt{\left(\sum X^{2}-\frac{{\left(\sum X\right)}^{2}}{M})(\sum Y^{2}-\frac{{\left(\sum Y\right)}^{2}}{M}\right)}}$$

### 2.2. Permutation Entropy

PE was proposed by Bandt and Pompe [43] to analyze the complexity of a time series and detect its dynamic characteristics. It has the advantages of low calculation cost and strong resistance to noise [34]. For a time series {x(i),i=1,2,...N}, the PE is calculated in the following steps.

1. Sequence reconstruction. The time series {x(i),i=1,2,...N} is converted into a multi-dimensional vector:
  (4) $$\left\{\begin{array}{l} X(1)=\{x(1),x(1+\tau ),...,x(1+(m-1)\tau )\}\\ \vdots \\ X(i)=\{x(i),x(i+\tau ),...,x(i+(m-1)\tau )\}\\ \vdots \\ X(N-(m-1)\tau )=\{x(N-(m-1)\tau ),x(N-(m-2)\tau ),...,x(N)\} \end{array}\right\}$$
  where *m* is the embedding dimension and τ is the delay factor.
2. Sorting. Each sequence in the embedding vector X(i) is rearranged in ascending order as: $x(i+k_{1})\tau )\leq x(i+k_{2}\tau )\leq ...\leq x(i+k_{m}\tau )$, where $k_{i}\neq k_{j}$. If two or more elements are equal, they are ordered by their corresponding index value $k_{i}$. The rank is the permutation of X(i), marked as an ordinal pattern $\pi (l)=\{k_{1},k_{2},k_{3},...,k_{m}\},l=1,2,3,...,m!$. There are in total *m*! possible ordinal patterns.
3. Probability and entropy calculation. The relative frequency of π(l) is calculated as:
  (5) $$P(\pi_{l})=\frac{\mathrm{number}\;\mathrm{of}\;\{X(i)\;\mathrm{has}\;\mathrm{type}\;\pi (l)\;|\;1\leq i\leq N-(m-1)\tau \}}{N-(m-1)\tau }$$
  The PE is defined as:
  (6) $$H_{PE}(m)=-\sum_{l=1}^{m!}P(\pi_{l})\ln P(\pi_{l})$$

The time lag τ and the embedding dimension *m* are recommended to be 1 and 3–7, respectively [29]. 

### 2.3. Improved Permutation Entropy

It can be seen from the definition of $P(\pi_{l})$ that the amplitude information is not taken into consideration. Vectors with a large difference, for example, {0, 1, 2}, {20, 21, 22} and {1, 12, 32} have the same motif (0, 1, 2), when *m* = 3. When calculating $P(\pi_{l})$, three vectors have the same weight of 1; therefore, their amplitude information is lost. In this study, we calculated Xr(i), which is the root-mean-square (RMS) value of X(i), and let it be the weight of X(i) to calculate the improved probability $P_{I}(\pi_{l})$:

(7) $$Xr(i)=\sqrt{\frac{\sum_{k=1}^{m}x^{2}(i+(k-1)\tau )}{m}}$$

(8) $$P_{I}(\pi_{l})=\frac{\;\sum {}_{i=1}^{N-(m-1)\tau }\{Xr(i)\;|X(i)\;\mathrm{has}\;\mathrm{type}\;\pi (l)\}}{\sum {}_{k=1}^{N-(m-1)\tau }\{Xr(k)\}}$$

Then, the IPE was then calculated by

(9) $$H_{IPE}(m)=-\sum {}_{l=1}^{m!}P_{I}(\pi_{l})\ln P_{I}(\pi_{l})$$

The definition of the IPE retains most of the PE’s properties and adds the amplitude information, so it is more accurate than the PE. A simulation contained impulse detection and signal segmentation was carried out to validate its advantage. The simulation signal was the combination of three component signals $y_{1}$, $y_{2}$, and $y_{3}$:

(10) $$\begin{array}{l} y_{1}(t)=\cos(4\pi t)\\ y_{2}(t)=6\cos(4\pi t)\\ y_{3}(t)=6\cos(8\pi t) \end{array}$$

**Figure 2:**
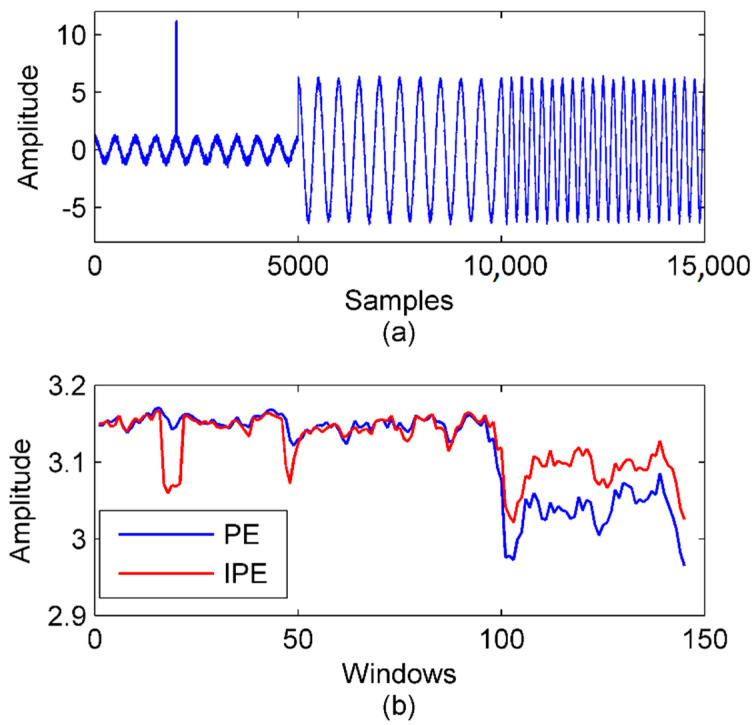
Application of the PE (permutation entropy) and IPE (improved permutation entropy) in impulse detection and signal segmentation. (**a**) The simulation signal. (**b**) The PE and IPE of the simulation signal with m=4,τ=1.

The length of each component signal was 1 s with 5000 samples, so there were 15,000 samples in total. $y_{1}$ and $y_{2}$ had the same frequency but different amplitudes. $y_{2}$ and $y_{3}$ had the same amplitude but different frequencies. An impulse was added to $y_{1}$ at 0.4 s. Additive white Gaussian noise (AWGN) with the signal-to-noise ratio (SNR) of 15 dB is also added to the whole signal. The wave of the simulation signal is shown in Figure 2a.

The PE and IPE of the simulation signal were calculated in sliding windows with a length of 500 samples. The window slid 100 samples per time. The curves of the PE and IPE are presented in Figure 2b. When the impulse occurred in windows 17–21, the IPE presented an obvious change, while the PE did not. That was because the PE does not consider amplitude information, so the impulse was treated the same as normal data. In signal segmentation, the boundary between $y_{2}$ and $y_{3}$ was detected by both the PE and IPE because the frequency change led to a noticeable change of the dynamic characteristic. However, the boundary between $y_{1}$ and $y_{2}$ was detected only by the IPE because the PE lost the amplitude information.

Therefore, the IPE proved to be more sensitive in detecting the dynamic characteristics of the signal than the PE.

### 2.4. PSO-SVM

The SVM method is based on the Vapnik-Chervonenkis (VC) dimension theory and the structural risk minimization principle. [44] It classifies two types by transforming the data to a higher dimensional feature space to find the optimal hyperplane in the space which maximizes the margin between the two types. Multi-classification can be solved by the combination of multiple binary SVMs, such as the “one-against-one” (OAO) and “one-against-all” (OAA) methods. A comparison showed that the OAO method is more suitable for practical use [45], so the OAO method was used here.

**Figure 3:**
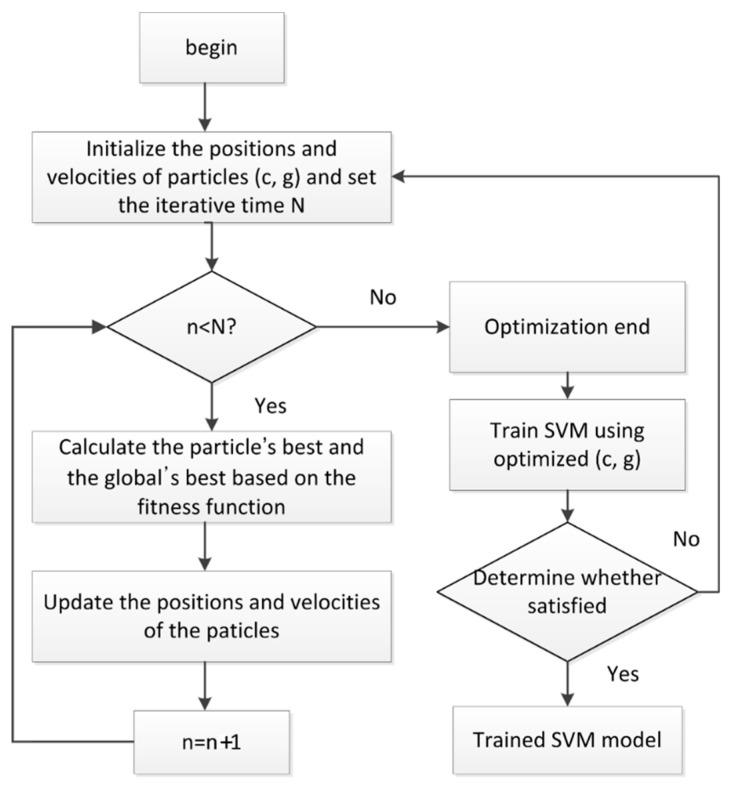
Flow chart of the PSO-SVM (particle swarm optimization–support vector machine).

The parameters in the SVM have a significant influence on the classification result. However, the parameter selection lacks theoretical guidance. The PSO is a computational intelligence method that is motivated by organisms’ behaviors, such as the flocking of birds. It has a well-balanced mechanism to enhance global and local exploration abilities. So the PSO was used to select the penalty parameter *c* and the kernel parameter *g* in the SVM with a Gaussian kernel. In the PSO, (*c*, *g*) become the particles. The PSO-SVM is briefly introduced as follows:

1. Initialize the particles (*c*, *g*) and the iterative time *N*.
2. Calculate the objective function value of the particle using the SVM training algorithm.
3. Calculate the optimal historical values of the individual and the population.
4. Update the particle velocity and position according to the speed and position update equations.
5. If the iterative time is satisfied, output the optimal parameters; otherwise, go back to step 3.
6. If the SVM accuracy does not meet the requirement, go back to step 1.
7. The flowchart of the PSO-SVM is shown in Figure 3.

### 2.5. Relationship Between Rope Vibration and Tension

Tension changes result in vibration changes of a wire rope. Vibration signals can be obtained from the drum and the head sheave. The drum connects to the gearbox and the motor, leading to serious noise and interferences. So the vibration signal is obtained from the head sheave. The wire rope between the head sheave and the drum can be seen as a taut string. In taut string theory, the relationship between the tension and the transverse frequency is described as [46]

(11) $$T=4\rho l^{2}{\left(\frac{f_{n}}{n}\right)}^{2}-\frac{EI}{l^{2}}{\left(n\pi \right)}^{2}$$

where T is the tension; ρ, l are the linear density and length of the rope between the head sheave and the drum, respectively; $f_{n}$ is the *n*-th order frequency of the rope, and EI is the bending stiffness of the rope. 

In mine hoisting, the bending stiffness of a rope is small and can be ignored [47]. So, the relationship can be simplified as

(12) $$T=4\rho l^{2}{\left(\frac{f_{n}}{n}\right)}^{2}$$

Although it seems simple, the transverse vibration frequency cannot be easily obtained by FT analysis because the frequency difference between the normal and fault states is very small. For instance, in a mine where ρ = 4 kg/m, l = 51 m and the base frequencies in the normal state $T_{1}$ = 45,000 N and the overload state $T_{2}$ = 50,000 N are 1.08 and 1.20 Hz, respectively, with a difference of 0.12 Hz. This means at least 8.3 s long data are needed to obtain high resolution in the FT method, which is unacceptable in terms of real-time performance. Further, some interference, such as wind, renders the signal nonlinear and nonstationary.

Therefore, the transverse vibration signal has a close relationship with rope tension and a novel processing method needs to be studied to extract useful features.

### 2.6. Proposed Method

**Figure 4:**
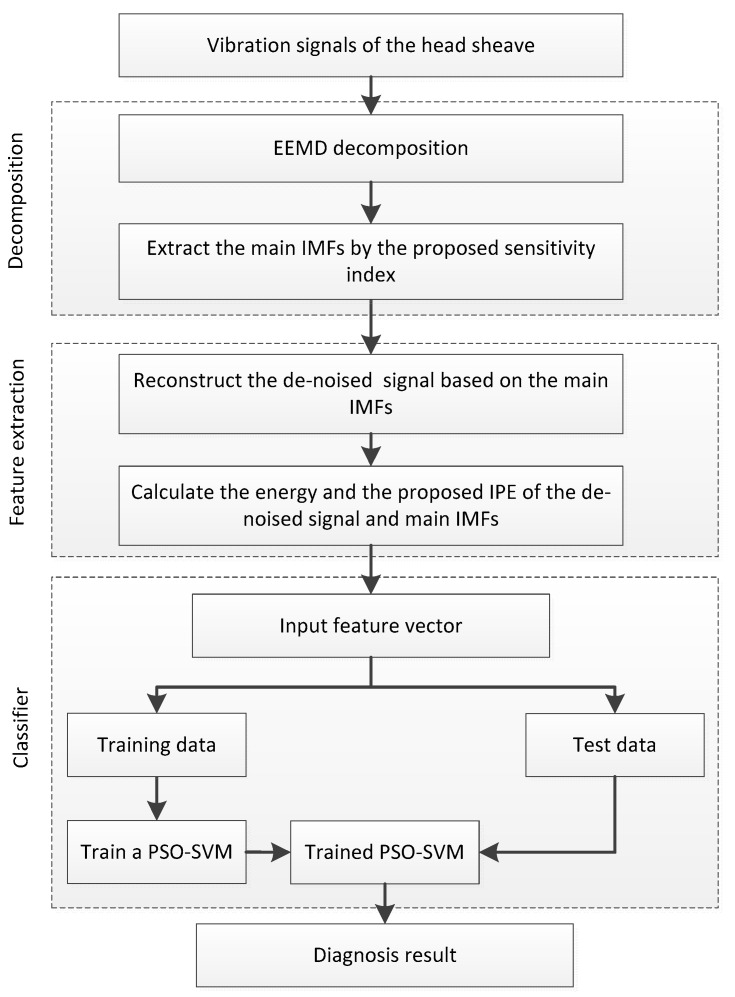
Flowchart of the proposed fault diagnosis method.

As is illustrated in Figure 4, the steps of the proposed method to diagnose the hoisting rope tension faults in a hoisting system are as follows.

1. Decompose the transverse vibration signal of the head sheave into IMFs by the EEMD method.
2. Calculate the proposed sensitivity index of the IMFs and sort the values in descending order; then, extract the top *j* IMFs as the main IMFs and reconstruct the de-noised signal by the sum of the main IMFs.
3. Calculate the energy and the IPE of the main IMFs and the de-noised signal to create the feature vector.
4. Train the PSO-SVM model based on the vibration samples in different tension states.
5. Use the trained SVM model to diagnose the rope tension faults in the hoisting systems in practice.

## 3. Experiment Results and Discussion

### 3.1. Experimental Setting and Data Collection

**Figure 5:**
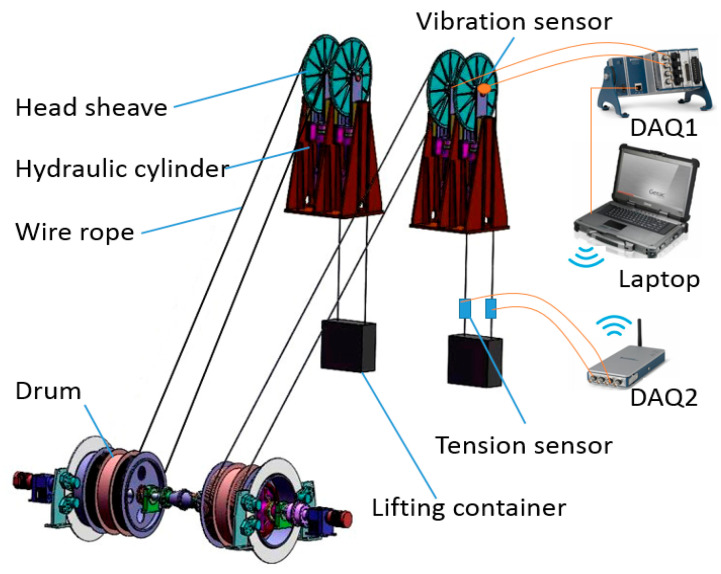
Schematic diagram of the two-rope winding hoist and the data acquisition system.

**Figure 6:**
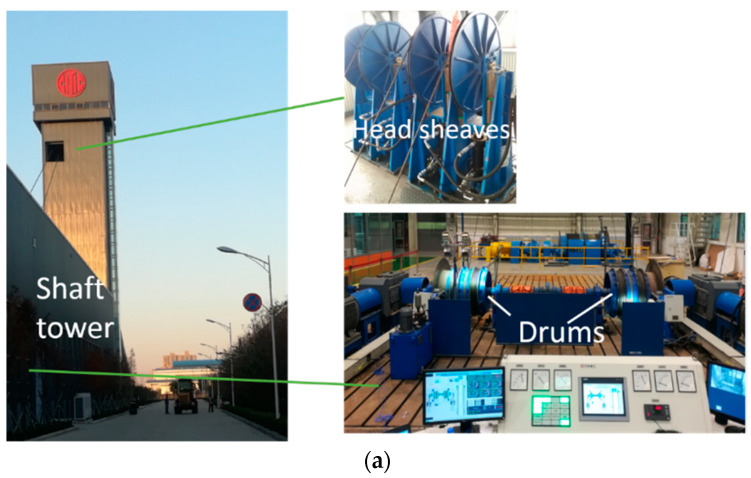

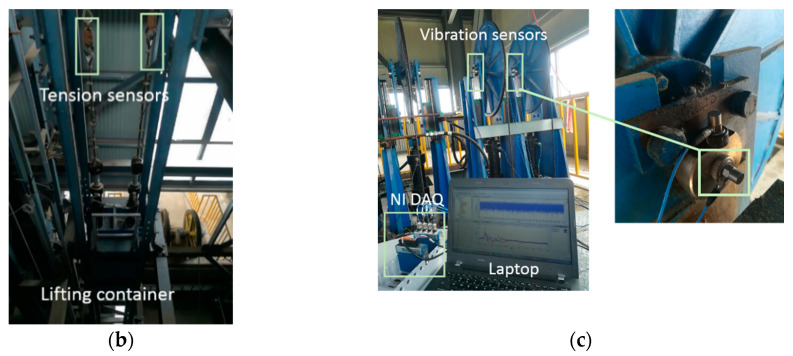
Photos of the experimental setup: (**a**) experimental platform, (**b**) tension sensors as calibration, and (**c**) vibration sensors and data acquisition system.

Rope tension faults include impact, overload, underload, and imbalance. Impact can be easily detected by a violent change of the amplitude. However, the other three states are difficult to classify because there are no essential differences among them, on which we focusedin this study. Fault experiments are very difficult to carry out in real mine hoists, so they were performed on an experimental platform. The experimental platform was a two-rope winding hoist with a lifting height of 36 m, the schematic diagram of which is shown in Figure 5. Two containers moved in opposite directions to balance the torque of the shaft apparatus. Each lifting container was connected to a winding drum by two parallel ropes. Each head sheave was fixed on a hydraulic cylinder, which could be adjusted by a control system. We used the hydraulic cylinders to create imbalance faults here. Photos of the platform are shown in Figure 6a. 

The experiment was performed on the two ropes of the right container. The schematic diagram of the sensors and the data acquisition system is also shown in Figure 5. Two ICP vibration sensors were independently attached to the end faces of the two head sheaves, as shown in Figure 6c. A National Instruments (NI) CompactDAQ data acquisition device and a laptop with LabVIEW software were used to acquire the vibration signal. Two tension sensors were installed between the right container and the two hoisting ropes. The tension data were obtained by another NI -CompactDAQ device that was placed in the container and were then transmitted to the laptop by wireless communication. The photos are shown in Figure 6b. Therefore, the transverse vibration data of the head sheaves and the ropes’ tension data were recorded at the same time by the LabVIEW software. The main parameters of the data acquisition system are shown in Table 1. The sampling frequency was set as 1024 Hz. 

In a single-rope hoist, there are three typical tension states called the overload, normal, and underload states. In a multi-rope hoist, the imbalance states are added. The five experimental tension states labeled S1-S5 are shown in Table 2, in which the two ropes are separately labeled as A and B. 

The overload and underload states were simulated by adjusting the weight of iron blocks in the lifting container. The imbalance state was simulated by adjusting the displacement of two hydraulic cylinders under the head sheaves. 

The lifting speed was set to be 1.5 m/s, and vibration and tension data were obtained in constant-speed lifting stages. 96 samples were obtained in each of the five states, so there were 480 samples in total. The sampling length was 1 s. 36 samples in each state were selected as the test samples and the others were selected as the training samples.

Two experiments were performed. Experiment 1 was the classification of the three states (S1–S3) of a single rope A, the aim of which was to present the advantages of each processing step of the proposed method by comparing it with other methods. Experiment 2 was the classification of the five states (S1–S5) of the two ropes, the aim of which was to show the generalization capability of the proposed method. 

### 3.2. Experiment 1: Classification of the Three States of a Single Rope 

#### 3.2.1. Signal Decomposition

**Figure 7:**
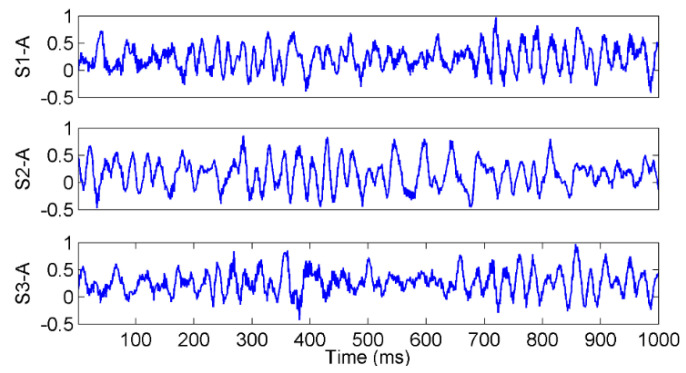
The waves of the vibration signals in different tension states.

**Figure 8:**
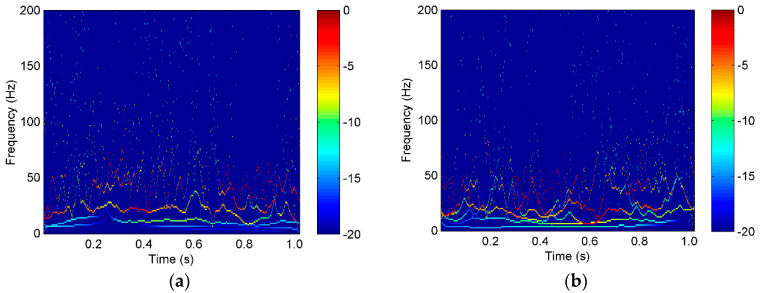

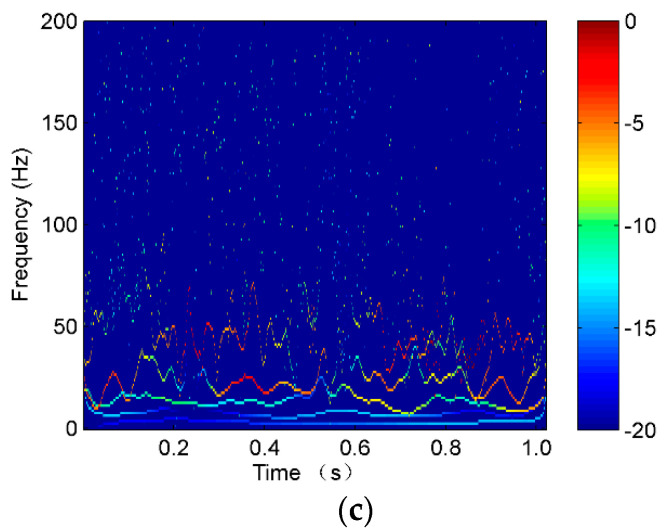
The Hilbert spectrum of the vibration waves of a single rope in the tension states of (**a**) S1-A, (**b**) S2-A, and (**c**) S3-A.

The vibration signal samples of rope A in the three states are shown in Figure 7, in which S1-A, S2-A, and S3-A are the overload, normal, and underload samples, respectively. The waves of the signal samples seem complicated. The energy distribution versus both the time and frequency of the signals is usually an effective feature to classify different running states, so the Hilbert spectra of the three states were calculated and are shown in Figure 8. As it can be seen, first, the energy was mainly distributed in the low frequency caused by the rope vibration, which means the noise caused by the motor was very small. Second, the distribution of the energy and frequency was not stable with time, which means the signals were time varying. This is because a hoist is an open system, in which there are many kinds of interference, such as wind. Third, it was difficult to find a clear difference among the spectra of the three states because they were quite complex. Further, we tried to use common time-domain statistical features, such as the mean, peak, RMS, and kurtosis values, to find the differences between the three states but also failed.

**Figure 9:**
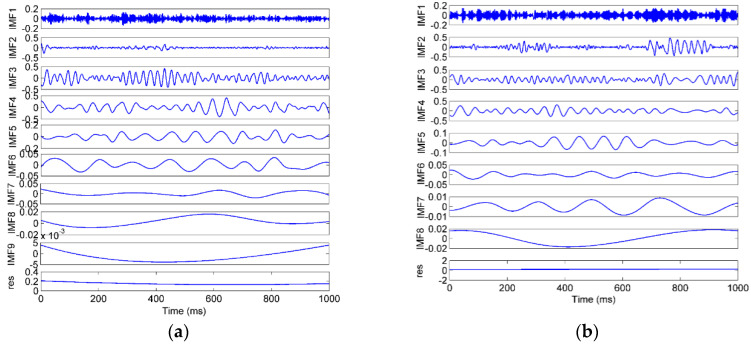
The decomposition result of a vibration sample by (**a**) EEMD (ensemble empirical mode decomposition), (**b**) EMD (empirical mode decomposition).

**Figure 10:**
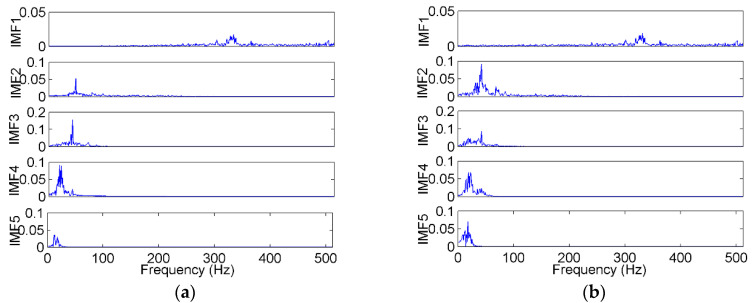
The FFT (fast Fourier transform) spectra of the IMFs (intrinsic module functions) decomposed by (**a**) EEMD and (**b**) EMD.

The signals were decomposed using the EEMD method, in which the amplitude of the added white noise was set to 0.2 times the standard deviation of the investigated signal, and the number of the ensembles was set to 50 [48]. The S2-A sample was used as an example, which was decomposed into several IMFs and a residue, as shown in Figure 9a. The EMD result of the same sample is shown in Figure 9b for comparison. The FFT spectra of the EEMD and EMD are shown in Figure 10. As shown in the time domain, the EMD and EEMD both decomposed the complicated raw signal into some simple components from high to low frequencies, which indicates the effectiveness of the EMD-based method. As shown in the frequency domain, in the EEMD results, each IMF’s frequency was more concentrated, and the frequencies between adjacent IMFs were more separate, especially in IMF2 and IMF3. That means that the mode mixing was improved by the EEMD approach.

**Figure 11:**
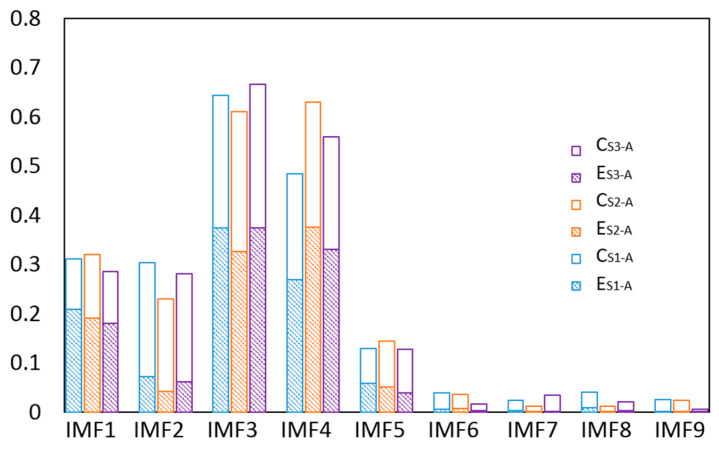
The sensitivity indexes of the IMFs.

The EEMD effectively decomposed the vibration signal into IMFs; however, not all IMFs were useful. The main IMFs were extracted to reduce noise based on the proposed sensitivity index in Equation (2), in which we regard the energy and the correlation to be equally important and set a = 0.5. The sensitivity index values of the IMFs are shown in Figure 11, in which *C* and *E* represent the relative correlation and relative energy, respectively. The top four IMFs were selected as the main IMFs. Then the de-noised signal was obtained by the sum of the main IMFs.

#### 3.2.2. Feature Extraction

**Figure 12:**
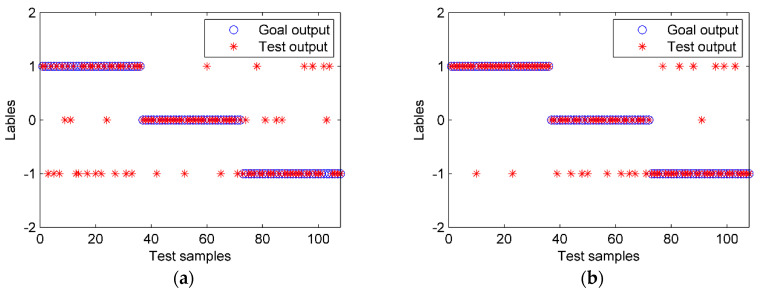

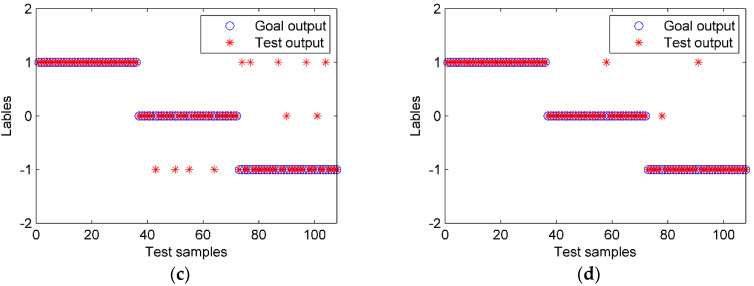
The PSO-SVM classification results with different features. (**a**) EN, (**b**) PE, (**c**) IPE, and (**d**) EN + IPE.

Feature extraction plays a crucial role in classification results. The energy and the IPE values of the IMFs and the de-noised signal were calculated, with the time lagτ and the embedding dimension *m* in the PE set to be one and four, respectively [20]. Six feature combinations were compared based on PE, IPE, and energy (EN), as shown in Table 3. The PSO-SVM was used as the classifier. The results are shown in Table 3 and Figure 12, in which the test samples 1–36, 37–72, 73–108 represent the S1-A, S2-A, and S3-A states, respectively.

We can observe the following information from the results: First, the accuracy of the IPE was much higher than that of the PE feature. That was because the IPE is more accurate, as it considers the amplitude of the signal. Second, combination features performed better than a single one because each feature has its advantage and multi-dimensional information is more comprehensive. For example, the EN was more sensitive to S3-A than to S1-A, while the IPE behaved in a contrary manner, and the combination of EN and IPE led to a much higher accuracy than the EN or IPE. Third, when all IMFs were used, the accuracy was much lower than when using the main IMFs and de-noised signal only. The reason for this is that some IMFs were interferences and noise, which indicates the necessity and effectiveness of the selection of the main IMFs. Last, the proposed feature extraction method had the highest accuracy of 97.2%.

#### 3.2.3. Optimized SVM

The OAO SVM was adopted to classify the overload, normal and underload states of a single rope. The PSO method was used to optimize the (*c*, *g*) parameters in the SVM with a Gaussian kernel. The five-fold cross-validation technique was used to improve the classification accuracy in the small sample problem. The feature vector was the IPE+EN of the main IMFs and the de-noised signal. The grid and GA methods were also applied for comparison. The results are shown in Table 4.

**Figure 13:**
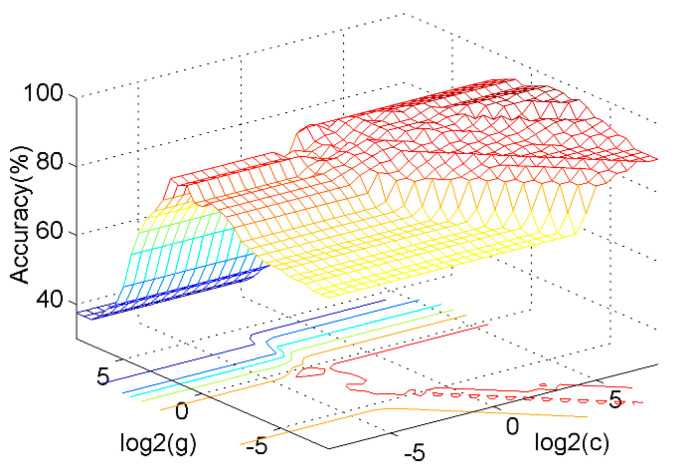
The influence of *c, g* parameters on the classification accuracy.

The result illustrates that the SVM model has the lowest classification accuracy. As shown in Figure 13, the *c*, *g* parameters have a significant influence on the classification accuracy. The accuracy may be as low as 40% based on a random selection, indicating the necessity of optimization. 

The grid, GA, and PSO algorithms increased the classification accuracy to 92.6%, 94.4%, and 97.2%, respectively. The computation time of the three methods was 7.4, 5.3, and 4.5 s, respectively. The grid algorithm is based on a traversal search that is highly time consuming when using a fine grid, while the GA and PSO are both population-based search methods that are usually time saving. Furthermore, the PSO takes less time than the GA because it is simpler and has no crossover and mutation processes. Therefore, the PSO works well to optimize the SVM in terms of both accuracy and computing time.

### 3.3. Experiment 2: Classification of the Five States of Multiple Ropes

The proposed signal decomposition, feature extraction, and classification methods were validated for the classification of the three tension states of a single rope. In a two-rope winding system, the feature vector’s dimension increases from five to ten. Moreover, the number of classifiers increases from three to ten because there are k(k−1)/2 classifiers in a *k*-class OAO SVM. So, the classification of the five states of two ropes is much more challenging. This experiment aimed to prove the generalization capability of the proposed method.

There were 300 training samples and 180 test samples. The features were extracted from the de-noised signal and the main IMFs of the two ropes. The fivefold cross-validation technique was also used to improve reliability. The results are shown in Table 5. It can be seen that the EEMD method performed better than the EMD at decomposition. The IPE performed better than the PE at feature extraction. The PSO-SVM performed better than the SVM and the neural network at classification. The comparison results are consistent with those from the classification of the single-rope hoist. The proposed method had the highest accuracy of 94.4%. Therefore, the results demonstrate the generalization capability of the proposed method and show its effectiveness for the tension fault classification in not only single-rope but also multi-rope hoists.

## 4. Conclusions

Rope tension faults may cause severe accidents in hoisting systems. In this paper, a novel tension fault diagnosis method based on the vibration signal of the head sheave was proposed. First, the signal was decomposed into several IMFs by the EEMD. Second, a sensitivity index was proposed to extract the main IMFs; then the de-noised signal was obtained by the sum of the main IMFs. Third, the IPE and the energy of the main IMFs and the denoised signal were calculated to create the feature vector. The IPE was proposed to improve the PE by adding the amplitude information of the signal, and it proved to be more sensitive than the PE in the simulations of impulse detecting and signal segmentation. Fourth, a PSO-SVM model was trained based on the vibration samples in different tension states. Lastly, the trained model was used to detect the tension faults in practice. Two experiments aiming at the classifications of the three states of a single rope and the five states of multiple ropes were performed. In the first experiment, the performances of different decomposition, feature extraction, and classification methods were independently compared to show the advantages of the proposed method. In the second experiment, the expansibility of the proposed method is verified. Results showed that the classification accuracies of the proposed method in the two experiments were 97.2% and 94.4%, respectively, which indicates that it can effectively detect rope tension faults such as overload, underload, and imbalance in hoisting systems. 

This study provides a new perspective for detecting rope tension faults in winding systems. Compared with the conventional method based on force sensors, the proposed method has obvious advantages: First, vibration sensors are much easier to install and do not affect the safety of the original structure. Second, data transmission and power supply are also much more convenient. Therefore, the proposed method has great potential for engineering applications.

The essence of tension fault diagnosis is the classification of the tension level of each rope. In this study, the classification of three tension levels of a single rope was realized. However, rope tension should be described more accurately in engineering by a classification of more levels or a regression analysis, which will be the topic of our future study. 

## Figures and Tables

**Table 1:** Main parameters of the data acquisition system.

	Module/Model	Main Parameters
Data acquisition 1	NI CompactDAQ with NI 9234 module	Sampling rate: 51.2 k/s; Resolution: 24 bit.
Data acquisition 2	NI CompactDAQ with NI-9220 module	Sampling rate: 100 k/s; Resolution: 16 bit.
Accelerometer	PCB 352C65	Measurement range: ±50 g; Frequency range: 0.5 Hz–10 kHz; Sensitivity: 100 mV/g.
Tension sensor	NOS-L101	Range: 10,000 N; Error: <1%.

**Table 2:** Setting of the experimental rope tension.

Label	State	The Tension of Rope A (N)	The Tension of Rope B (N)
S1	Overload	5600	5600
S2	Normal	4900	4900
S3	Underload	4200	4200
S4	Imbalance 1	5600	4200
S5	Imbalance 2	4200	5600

**Table 3:** Classification results using different features.

Feature	IMFs	S1-A Accuracy (%)	S2-A Accuracy (%)	S3-A Accuracy (%)	Total Accuracy (%)
EN	De-noised signal and main IMFs	61.1	86.1	72.2	73.1
PE		94.4	75.0	80.6	83.3
IPE		100.0	88.9	80.6	89.8
{PE, EN}		100.0	94.4	88.9	94.4
{IPE, EN}		100.0	97.2	94.4	97.2
{IPE, EN}	All IMFs	88.9	88.9	86.1	88.0

**Table 4:** Classification results using different SVM (support vector machine) models.

Model	Total Accuracy (%)	Computing Time(s)
SVM	88.0	0.1
Grid-SVM	92.6	7.4
GA-SVM	94.4	5.3
PSO-SVM	97.2	4.5

**Table 5:** Classification results of rope tension states of the two ropes.

Decomposition	Feature Extraction	Classification Model	Total Accuracy (%)
EMD	IPE + EN	PSO-SVM	83.3
EEMD	IPE + EN	PSO-SVM	94.4
EEMD	IPE + EN	BP neural network	82.2
EEMD	IPE + EN	SVM	78.9
EEMD	PE + EN	PSO-SVM	92.2

## Abbreviations

ANN	artificial neural network
AWGN	additive white Gaussian noise
DL	deep learning
EEMD	ensemble empirical mode decomposition
EMD	empirical mode decomposition
EN	energy
FT	Fourier transform
GA	genetic algorithm
IMF	intrinsic module function
IPE	improved permutation entropy
kNN	k Nearest Neighbor
NI	National Instruments
OAA	one-against-all
OAO	one-against-one
PE	permutation entropy
PSO	particle swarm optimization
RMS	root-mean-square
SNR	signal-to-noise ratio
STFT	short-time Fourier transform
SVM	support vector machine
VC	Vapnik-Chervonenkis
WT	wavelet transform
